# Advancing Psoriasis Care through Artificial Intelligence: A Comprehensive Review

**DOI:** 10.1007/s13671-024-00434-y

**Published:** 2024-06-13

**Authors:** Payton Smith, Chandler E. Johnson, Kathryn Haran, Faye Orcales, Allison Kranyak, Tina Bhutani, Josep Riera-Monroig, Wilson Liao

**Affiliations:** 1 Department of Dermatology, University of California San Francisco, San Francisco, CA, USA; 2 Dermatology Department, Hospital Clínic de Barcelona, Universitat de Barcelona, Barcelona, Spain

**Keywords:** Machine learning, Dermatology, Precision medicine, Psoriasis

## Abstract

**Purpose of Review:**

Machine learning (ML), a subset of artificial intelligence (AI), has been vital in advancing tasks such as image classification and speech recognition. Its integration into clinical medicine, particularly dermatology, offers a significant leap in healthcare delivery.

**Recent Findings:**

This review examines the impact of ML on psoriasis—a condition heavily reliant on visual assessments for diagnosis and treatment. The review highlights five areas where ML is reshaping psoriasis care: diagnosis of psoriasis through clinical and dermoscopic images, skin severity quantification, psoriasis biomarker identification, precision medicine enhancement, and AI-driven education strategies. These advancements promise to improve patient outcomes, especially in regions lacking specialist care. However, the success of AI in dermatology hinges on dermatologists’ oversight to ensure that ML’s potential is fully realized in patient care, preserving the essential human element in medicine.

**Summary:**

This collaboration between AI and human expertise could define the future of dermatological treatments, making personalized care more accessible and precise.

## Introduction

Machine learning (ML) refers to a set of algorithms that can perform tasks with human-like cognitive abilities [1]. It utilizes statistical techniques to enable software applications to learn from and adapt to training data by adjusting their parameters based on the data encountered [1]. The advent of this technology has revolutionized tasks like image classification and speech recognition since 2012 [1]. Integrating machine learning into clinical medicine presents a promising opportunity to markedly enhance the delivery of healthcare services [2]. Today, artificial intelligence (AI) is being explored in medicine to improve diagnostics, healthcare accessibility, and patient outcomes [3]. In dermatology, AI introduces distinct benefits by mitigating the subjectivity inherent in evaluating dermatosis severity, a domain where dermatologists predominantly depend on visual assessments more than other medical fields. This aspect is particularly crucial in managing psoriasis, where quantifying the affected body surface area (BSA) or utilizing scales like the Psoriasis Area and Severity Index (PASI) and Physician Global Assessment (PGA) can determine a patient’s eligibility for transformative treatments. Consequently, AI stands to significantly improve the accuracy of diagnoses and the customization of treatments for conditions like psoriasis, marking a new era of precision in dermatological care.

This review delves into the current state of ML applications pertinent to dermatologists, focusing on psoriasis and identifying five key areas where ML is making significant strides in dermatology. These include (1) classifying psoriasis using clinical and dermatopathology images, (2) quantifying psoriasis severity using machine learning, (3) leveraging AI for the discovery of psoriasis biomarkers, (4) enhancing precision medicine for psoriasis patients, and (5) AI-enhanced education and engagement strategies. Furthermore, we address potential obstacles and the future scope for ML advancements in this field.

## Utilizing AI Algorithms to Diagnose Psoriasis Via Image Analysis

Introducing ML into dermatology, particularly through Convolutional Neural Networks (CNNs), represents a pivotal shift in diagnosing psoriasis. CNNs — advanced deep learning models developed to detect and learn features from structured grid data like images — have demonstrated initial success in recognizing psoriasis from both clinical and dermoscopic images. This offers a promising solution to overcome the limitations faced in areas with few dermatologists or where practitioners may have limited dermatological expertise.

Research highlights the effectiveness of CNNs in accurately diagnosing psoriasis, outperforming both traditional models and expert dermatologists in some cases. A 2019 study by Zhao et al. showcased a two-stage deep-learning model that exceeded the diagnostic accuracy of 25 Chinese dermatologists [4]. Similarly, Yang et al.’s work with CNNs on dermoscopic images of psoriasis revealed high sensitivity and specificity, equating to the capabilities of board-certified dermatologists [5]. Moreover, Yu et al. developed a CNN model that distinguished between scalp psoriasis and seborrheic dermatitis more accurately than dermatologists, highlighting AI’s potential to improve diagnostic accuracy and support clinical decision-making [6].

A major challenge in diagnosing psoriasis with machine learning is accurately identifying the boundaries of skin lesions. A 2020 study by Dash et al. addressed this by combining newer algorithms with traditional methods. They used advanced techniques, such as seeker optimization and particle swarm optimization, along with traditional clustering methods like K-means and fuzzy C-means. The study found that combining fuzzy C-means with seeker optimization was the most effective. This combined method significantly improved the accuracy of identifying psoriasis lesions by finding the best central points for grouping similar areas of skin, also known as optimizing cluster centroids. As a result, it could better distinguish between healthy skin and psoriasis lesions, leading to more precise identification [7].

## Psoriasis Severity Quantification Through AI

The severity of plaque psoriasis is often defined by the extent of the disease (BSA) and intensity of the lesions (measured by PASI) [8]. It has been noted that PASI scores have considerable intra- and interobserver variability [9]. To address this issue and strive for a more precise measurement of the severity of plaque psoriasis, especially in clinical trials, various alternative or enhancing methods have been explored. These methods include efforts to automatically measure the erythema of psoriasis plaques or calculate severity with PASI using photographs and eventually utilizing artificial intelligence and the application of deep learning algorithms [10, 11].

BSA calculation using machine learning was accurate in more than 90% of cases and differed only 5.9% from previously marked areas for dermatologists [12]. The largest study performed in automated PASI calculation, which included more than 14,000 images and involved 43 dermatologists, showed that the proposed image-AI model outperformed the average performance of dermatologists with a 33.2% performance gain in the overall PASI score using mean absolute error as an indicator of accuracy [13]. The AI model functioned best with access to more photographs and less severe (lower PASI score) cases and was able to predict the trend of severity in patients who improved or regressed for 84.81% of cases.

AI in psoriasis severity calculation in clinical practice and teledermatology has also been explored. Okamoto and colleagues developed an automated single-shot assessment of psoriasis severity using photographs of the trunk [14]. This freely available software allows the upload of images and calculation of severity.

Some locations that may be affected by psoriasis, such as the nails, palms, and soles, present challenges for plaque psoriasis severity calculations. Two studies have explored the usefulness of AI in aiding in the evaluation of psoriasis severity in these body regions. Nail psoriasis assessment through the nail psoriasis severity index (NAPSI) requires expertise and is time-consuming [15]. In contrast to PASI, NAPSI is not routinely used in clinical practice. Modified NAPSI calculation of fingernails using deep learning showed a good performance with an area under the receiving operator curve (AUROC) of 88% [16].

One of the main limitations of PASI and BSA automated calculation is that the images used to train the algorithm usually do not include palmoplantar lesions. In the case of palmoplantar pustulosis, a variant of pustular psoriasis, the score used is Palmoplantar Pustular Psoriasis Area and Severity Index (PPPASI). Deep learning algorithms have been developed to overcome the inconsistency between different graders of PPASI, showing a good interclass correlation coefficient of 0.879 and low differences in the percentage of affected area [17].

Apart from the limitations described previously, the assessment of induration with AI tools, the measurement of scalp psoriasis severity and the applicability in both clinical trials and practice remain to be explored.

## Utilizing AI to Identify Biomarkers in Psoriasis

There is a critical need for the discovery of psoriasis-associated biomarkers, as they may facilitate diagnosing psoriasis, monitoring disease activity, predicting treatment response, and predicting the occurrence of co-morbidities. The use of AI for biomarker discovery has been on the rise due to the accumulation of high-dimensional data with thousands of variables, a common one being RNA sequencing data, which contains the sequencing profile of thousands of genes [18, 19].

RNA sequencing data has been most commonly used with AI to identify potential genomic biomarkers [20–23]. In the studies by Yao et al., Xing et al., and Liu et al., multiple ML models were used to identify diagnostic biomarkers through computed feature importance. The most important features (diagnostic biomarkers) from each model would be compared to identify overlap. Yao et al. found that ADAM23 was highly expressed in psoriasis lesions compared to healthy controls, Xing et al. found that GJB2 was also significantly overexpressed, and Liu et al. discovered three overlapping biomarkers, IRS1, RAI14, and ARH-GEF10, with lower expression levels in psoriasis lesions compared to normal controls [20, 22, 23]. Deng et al.’s study similarly used feature importance to identify biomarkers, but instead of computing feature importance for multiple models, their study used a Random Forest (RF) model to identify core biomarkers and then verified the performance of these biomarkers using a Support Vector Machines (SVM) model. This study, in particular, found that PI3 was highly expressed in psoriatic skin and was positively correlated with disease severity [21]. Song et al.’s study identified various pyroptosis-related biomarkers for diagnosing psoriasis using features with the highest importance [24]. Similar to Deng et al.’s study, Song et al. used an RF model to identify core biomarkers. However, these biomarkers were not verified with other ML models. Across these five sequencing-based studies, the most common and reliable ML models used were RF and SVM.

More recently, imaging and metabolomic data have been used with AI to identify potential biomarkers [25–27]. He et al. used a deep learning ML model on three-dimensional ultra-wideband raster-scan optoacoustic mesoscopy (RSOM) images. This study looked at imaging biomarkers and found that PASI values are closely related to epidermal thickness [25]. Choksi et al. and Koussiouris et al. used metabolomic data to find biomarkers associated with skin disease activity levels in psoriatic arthritis (PsA) patients. Liu et al. used single-cell RNA-seq from peripheral blood mononuclear cells to diagnose psoriatic arthritis [28]. All three studies also identified biomarkers using the feature importance method and narrowed it down to several core biomarkers that overlapped between different ML models. Choksi et al. identified various metabolic markers associated with skin disease activity in PsA patients, including various metabolites involved in fatty acid metabolism and immune-mediated pathways [26]. Koussiouris et al. also found several significant metabolites associated with disease activity levels in PsA patients, including the presence of lipids such as lyphosphatidylcholine and sphingomyelin [27].

All of these studies presented results from well-performing models (AUROC > 0.8). For PsA, there are currently no clinically validated biomarkers. ML is becoming a promising method for identifying potential biomarkers to ultimately improve diagnostic tools and patient-specific responses to treatment. However, none of them have yet been validated in clinical practice.

## Role of AI in Personalized Care and Enhancing Psoriasis Treatment

When deciding to prescribe psoriasis treatment beyond topicals, clinicians are challenged to determine what other treatment modalities to use, how to quantify patient response, and how to limit trial-and-error prescribing. Recent research has focused on utilizing AI to enhance and personalize psoriasis treatment.

In patients with insufficient response to topical therapies, phototherapy is often the next step in the treatment algorithm [29]. However, it is time-consuming and inaccessible to many patients [30]. As such, research has focused on predicting individual patient responses to phototherapy to determine if it is a reasonable treatment option. For example, Narbutt et al. used machine learning models to predict 82 psoriasis patients’ responses to narrow-band ultraviolet B (NB-UVB) phototherapy [29]. They used questionnaires and standard blood tests to create a random forest classifier with 84% sensitivity and predicted a short remission and good outcome with 85% and 75% accuracy, respectively. Unfortunately, they had a significant loss of follow-up (70%), and their research only included NB-UVB therapy. However, due to the ease of administering patient questionnaires and the use of standardized labs in creating their classifiers, Narbutt et al. propose a clinically translational use of AI in phototherapy treatment.

Significant research has also focused on creating ML classifiers that predict patient response to systemic therapies. In their proof-of-concept study, Foulkes et al. used ML to predict change in PASI in 10 patients with severe chronic plaque psoriasis initiating etanercept [31]. They focused on developing classifiers across multiple platforms (mRNA vs. miRNA) and tissue types (tissue vs. blood). Other studies have used retrospective data from clinical trials to develop machine learning classifiers that accurately predict patient response to systemic therapies, such as Correa da Rosa, who predicted week 12 response with 97% accuracy following one to two weeks of therapy [32, 33]. Bagel et al. took this one step further, using classifiers developed from published clinical trial data, testing their dermal diagnostic patch to extra mRNA, and predicting therapy response from the psoriatic skin samples [34]. Du et al. took a slightly different approach and, using data from a Danish registry and various machine learning algorithms, predicted the likelihood of biologic discontinuation within five years with an accuracy ranging from 65.3% to 77.5% and determined what factors–patient sex, body weight, and drug type-were most predictive of discontinuation [35].

AI has also been utilized to measure patient response to therapies. Orsini et al. highlighted this by using AI to segment line-field confocal optical coherence tomography (LC-OCT) images of the skin in Italian patients with psoriasis treated with various therapies [36]. In a sample size of 17, they found a significant correlation between clinical lesion score and epidermal and stratum corneum thickness, thus demonstrating the ability to supplement clinical qualitative assessments with AI-derived quantitative mechanisms.

## Helping Psoriasis Patients Through AI-driven Education and Engagement

There is a gap in the literature on the topic of psoriasis- and psoriatic arthritis-specific AI-driven education and engagement. However, in a broader sense, the field of dermatology has shown promising growth in this area in recent years. Much of the work in this area shares the commonality of the use of a variety of ChatGPT versions or chatbots for the purpose of various patient education modalities. The use of this technology has been shown to have utility ranging from responding to questions posed through a patient portal to creating original patient-facing educational materials [37, 38].

Now more than ever, clinicians spend a significant amount of time completing documentation following patient encounters—including initiating prior authorizations and responding to patient inquiries via a patient portal. Based on a 2024 study by Reynolds et al., one notable advantage of the use of AI in this context is the potential to save time for providers by developing a first draft of such correspondences [37]. This study also evaluated patient portal message questions and the responses that had been provided by the patients’ dermatologists. Those same patient questions were posed to ChatGPT (Version 3.5). The ChatGPT output responses and the original dermatologist responses were compared by ten blind reviewers for overall quality, readability, accuracy, thoroughness, and level of empathy. Reviewers preferred the responses by the dermatologists over those generated by ChatGPT, citing increased readability and level of empathy. However, it is important to note that these are stylistic rather than content-related concerns. Specifically, there were no instances of hallucinations within the ChatGPT responses to these patient inquiries. This refers to instances where incorrect information is generated by a chatbot when there is a lapse in the content that is necessary to draw a conclusion.

A related advantage of the use of AI in this context is to enhance providers’ timeliness in communicating laboratory or dermatopathology results to patients. In addition to the option to communicate directly with their dermatologist, the patient portal often allows patients immediate access to dermatopathology reports. Such reports contain medical jargon and generally require interpretation by the patient’s dermatologist. A 2024 study by Zhang et al. revealed that dermatopathology reports translated into patient-friendly language by ChatGPT-4 were complete, accurate, understandable, and unlikely to cause harm [39]. This study reiterates the potential for chatbots to serve as a resource for clinicians to enhance their efficiency in providing accurate responses and interpretations of results in patient-friendly language.

It is important that dermatologists consider the health literacy of their patients, as well as the use of patient-friendly language in patient-doctor interactions. By doing so, dermatologists facilitate opportunities for patients to become more active participants in their plan of care. A 2023 study by Mondal et al. demonstrated that ChatGPT could produce easily understandable patient education materials at an appropriate Flesh-Kincaid Grade Level for a list of fourteen of the most-queried dermatological conditions [38]. This promising finding reveals the potential for accurate, appropriate, tailored educational materials for common dermatologic conditions.

Though the previously mentioned AI-driven education and engagement initiatives show promise, there are some disadvantages. The quality of the responses generated by ChatGPT and other chatbots relies upon the quality of the data that it draws upon, which may be outdated or simply incorrect. The data may also be incomplete, which may lead to hallucinations. In addition, there is a risk of content plagiarism, particularly during the creation of patient-facing educational materials. These disadvantages have the potential to harm patients via misinformation. Goodman et al. astutely summarize: “Even a 99.9% safety rate is unacceptable” [38]. Therefore, materials and responses generated by ChatGPT and other chatbots should, in most cases, incorporate a safety checkpoint in the form of clinician evaluation of content before release to a patient.

Going forward, further attention must be allotted to psoriasis- and psoriatic arthritis-specific AI-driven education and engagement. There is ample opportunity for developments that have the potential to enhance the patient experience, as well as health outcomes.

## Dataset Biases, Limitations, and Need for Future Research

Despite these advancements, the application of AI in dermatology faces challenges, particularly in terms of inclusivity and transparency concerning the datasets used for training. The underrepresentation of diverse skin tones and the suboptimal performance of AI across different demographics underscore the need for unbiased AI tools. For instance, the study by Zhao et al. utilized a dataset primarily comprising images from Chinese patients, and the studies by Yang et al. and Strober et al. did not provide detailed demographic information about the patients in the dataset, which could affect the model’s generalizability to different demographic groups. Studies by Chen et al. and Daneshjou et al. emphasize the importance of creating AI algorithms that can diagnose skin diseases accurately across a varied patient population, ensuring equitable healthcare delivery [40, 41]. To further mitigate these biases, future research must focus on developing and validating AI tools using diverse datasets that encompass various skin tones, ages, and geographic regions. Collaborative efforts to create large, inclusive databases will be crucial in enhancing the fairness and accuracy of AI applications in dermatology.

## Conclusion

**Fig. 1:**
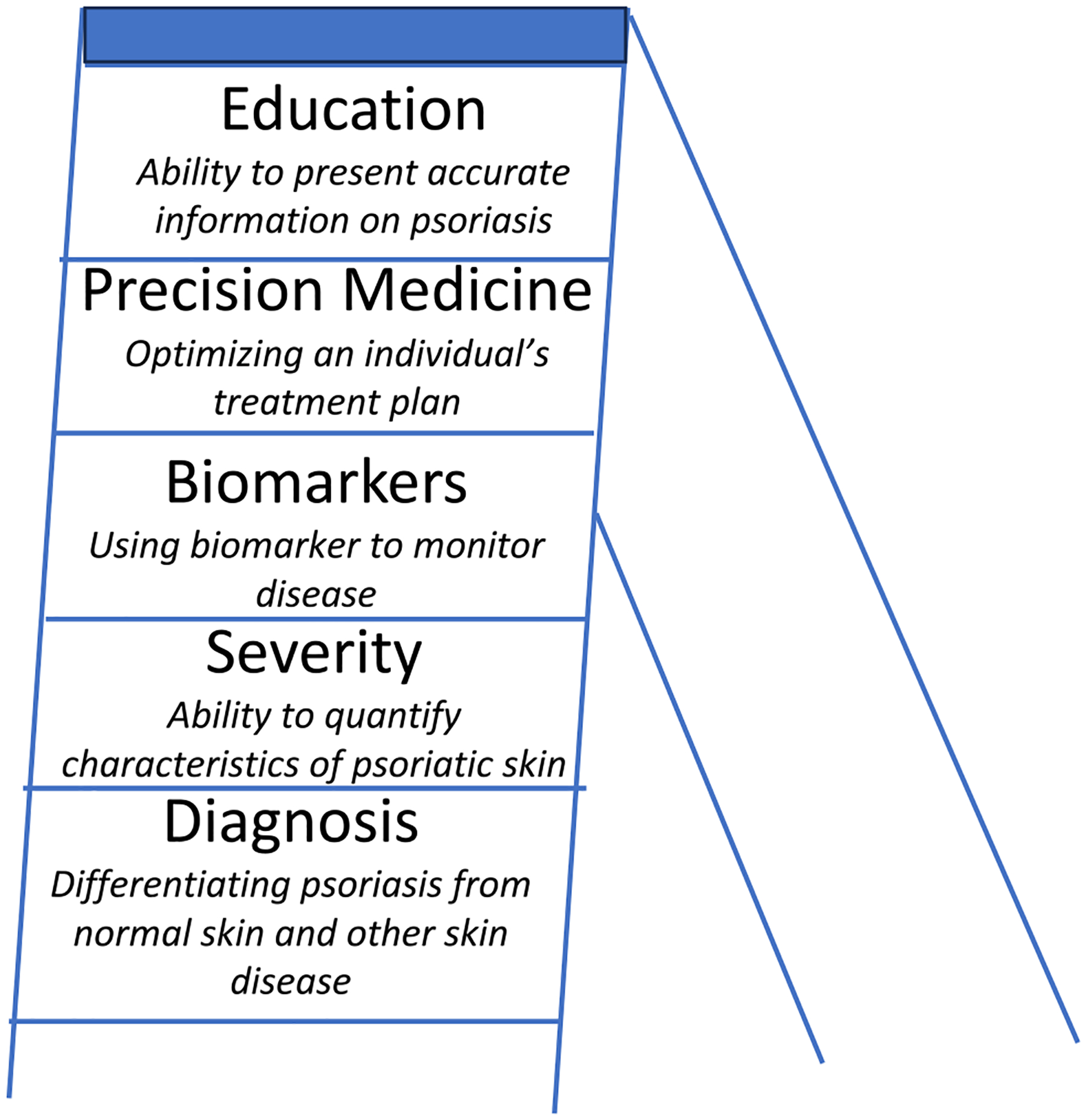
Ladder schematic detailing potential uses for AI in psoriasis. The ability of AI to diagnose psoriasis (1st run of ladder) is important for AI to potentially quantify psoriasis severity (2nd rung) and utilize biomarkers to track psoriasis progression (3rd rung). With these accomplishments, one could then feasibly use an AI system to suggest therapies for patients based on their severity and biomarkers (4th rung). If an AI system has a comprehensive understanding of psoriasis, AI could then educate patients and practitioners (top rung)

As the field of dermatology advances, clinical integration of AI shows exciting promise for enhancing the precision and personalization of medicine, especially for psoriasis patients. This advancement is invaluable, particularly in areas underserved by specialist dermatological care. As seen in Fig. 1, AI can play a significant role across various stages of psoriasis management, from initial diagnosis to tasks like recommending treatments and educating patients. However, the effectiveness of AI tools, much like traditional clinical instruments, relies heavily on the careful supervision of dermatologists, ensuring that the promise of machine learning translates into enhanced, personalized patient care. This approach not only maximizes the potential of AI to enhance treatment outcomes but also maintains the indispensable human touch in healthcare.

## Data Availability

No datasets were generated or analysed during the current study.

## References

[1] Thomsen K, Iversen L, Titlestad TL, Winther O. “Systematic review of machine learning for diagnosis and prognosis in dermatology”, (in eng). J Dermatolog Treat. 2020;31(5):496–510. 10.1080/09546634.2019.1682500. 31625775

[2] Char DS, Shah NH, Magnus D. “Implementing machine learning in health care - addressing ethical challenges,” (in eng). N Engl J Med. 2018;378(11):981–3. 10.1056/NEJMp1714229. 29539284 PMC5962261

[3] Aminizadeh S, “Opportunities and challenges of artificial intelligence and distributed systems to improve the quality of healthcare service”, (in eng). Artif Intell Med. 2024;149: 102779. 10.1016/j.artmed.2024.102779. 38462281

[4] Zhao S, “Smart identification of psoriasis by images using convolutional neural networks: a case study in China”, (in eng). J Eur Acad Dermatol Venereol. 2020;34(3):518–24. 10.1111/jdv.15965. 31541556

[5] Yang Y, “A convolutional neural network trained with dermoscopic images of psoriasis performed on par with 230 dermatologists”, (in eng). Comput Biol Med. 2021;139: 104924. 10.1016/j.compbiomed.2021.104924. 34688173

[6] Yu Z, Kaizhi S, Jianwen H, Guanyu Y, Yonggang W. A deep learning-based approach toward differentiating scalp psoriasis and seborrheic dermatitis from dermoscopic images, (in eng). Front Med (Lausanne). 2022;9:965423. 10.3389/fmed.2022.965423. 36405606 PMC9669613

[7] Dash M, Londhe ND, Ghosh S, Shrivastava VK, Sonawane RS. “Swarm intelligence based clustering technique for automated lesion detection and diagnosis of psoriasis”, (in eng). Comput Biol Chem. 2020;86: 107247. 10.1016/j.compbiolchem.2020.107247. 32413831

[8] Strober B, “Recategorization of psoriasis severity: delphi consensus from the international psoriasis council,” (in eng). J Am Acad Dermatol. 2020;82(1):117–22. 10.1016/j.jaad.2019.08.026. 31425723

[9] Wu AG, Conway J, Barazani L, Roy B, Cline A, Pereira F. “Is clear always clear? comparison of psoriasis area and severity index (pasi) and the physician’s global assessment (PGA) in psoriasis clearance,” (in eng). Dermatol Ther (Heidelb). 2020;10(5):1155–63. 10.1007/s13555-020-00435-2. 32804321 PMC7477033

[10] Raina A, “Objective measurement of erythema in psoriasis using digital color photography with color calibration”, (in eng). Skin Res Technol. 2016;22(3):375–80. 10.1111/srt.12276. 26517973 PMC4851905

[11] Fink C, Alt C, Uhlmann L, Klose C, Enk A, Haenssle HA. “Precision and reproducibility of automated computer-guided Psoriasis Area and Severity Index measurements in comparison with trained physicians”, (in eng). Br J Dermatol. 2019;180(2):390–6. 10.1111/bjd.17200. 30218575

[12] Meienberger N, “Observer-independent assessment of psoriasis-affected area using machine learning”, (in eng). J Eur Acad Dermatol Venereol. 2020;34(6):1362–8. 10.1111/jdv.16002. 31594034

[13] Huang K, Artificial intelligence-based psoriasis severity assessment: real-world study and application (in eng). J Med Internet Res. 2023;25:e44932. 10.2196/44932. 36927843 PMC10131673

[14] Okamoto T, Kawai M, Ogawa Y, Shimada S, Kawamura T. “Artificial intelligence for the automated single-shot assessment of psoriasis severity”, (in eng). J Eur Acad Dermatol Venereol. 2022;36(12):2512–5. 10.1111/jdv.18354. 35739649

[15] Aggarwal P, “Clinical characteristics and disease burden in prurigo nodularis”, (in eng). Clin Exp Dermatol. 2021;46(7):1277–84. 10.1111/ced.14722. 33969517

[16] Folle L, “DeepNAPSI multi-reader nail psoriasis prediction using deep learning”, (in eng). Sci Rep. 2023;13(1):5329. 10.1038/s41598-023-32440-8. 37005487 PMC10067940

[17] Paik K, Kim BR, Youn SW. “Evaluation of the area subscore of the palmoplantar pustulosis area and severity index using an attention U-net deep learning algorithm,” (in eng). J Dermatol. 2023;50(6):787–92. 10.1111/1346-8138.16752. 36815336

[18] Liu Z, Wang X, Ma Y, Lin Y, Wang G. “Artificial intelligence in psoriasis: Where we are and where we are going”, (in eng). Exp Dermatol. 2023;32(11):1884–99. 10.1111/exd.14938. 37740587

[19] Hong J, Mosca M, Hadeler E, Hakimi M, Bhutani T, Liao W. The future of personalized medicine in psoriasis. Dermatol Rev. 2021;2:282–8. 10.1002/der2.87.

[20] Yao P, “Identification of ADAM23 as a potential signature for psoriasis using integrative machine-learning and experimental verification,” (in eng). Int J Gen Med. 2023;16:6051–64. 10.2147/IJGM.S441262. 38148887 PMC10750783

[21] Deng J, “Multi-omics approach identifies PI3 as a biomarker for disease severity and hyper-keratinization in psoriasis”, (in eng). J Dermatol Sci. 2023;111(3):101–8. 10.1016/j.jdermsci.2023.07.005. 37543503

[22] Xing L, “Exploration of biomarkers of psoriasis through combined multiomics analysis,” (in eng). Mediators Inflamm. 2022;2022:7731082. 10.1155/2022/7731082. 36193416 PMC9525798

[23] Liu Y, Cui S, Sun J, Yan X, Han D. Machine learning analysis of human skin by optoacoustic mesoscopy for automated extraction of psoriasis and aging biomarkers. IEEE Trans Med Imag 2024. 10.1109/TMI.2024.3356180. Epub ahead of print. 38241120

[24] Song JK, “Classification and biomarker gene selection of pyroptosis-related gene expression in psoriasis using a random forest algorithm”, (in eng). Front Genet. 2022;13: 850108. 10.3389/fgene.2022.850108. 36110207 PMC9468882

[25] He H Machine learning analysis of human skin by optoacoustic mesoscopy for automated extraction of psoriasis and aging biomarkers (in eng). IEEE Trans Med Imag. 2024. 10.1109/TMI.2024.3356180. 38241120

[26] Choksi H, Identifying serum metabolomic markers associated with skin disease activity in patients with psoriatic arthritis (in eng), Int J Mol Sci. 2023;24(20). 10.3390/ijms242015299. PMC10607811 37894979

[27] Koussiouris J, Looby N, Kotlyar M, Kulasingam V, Jurisica I, Chandran V. Classifying patients with psoriatic arthritis according to their disease activity status using serum metabolites and machine learning (in eng). Metabolomics. 2024;20(1):17. 10.1007/s11306-023-02079-7. 38267619 PMC10810020

[28] Liu J, Combined single cell transcriptome and surface epitope profiling identifies potential biomarkers of psoriatic arthritis and facilitates diagnosis (in eng). Front Immunol. 2022;13. 10.3389/fimmu.2022.835760 PMC8924042 35309349

[29] Narbutt J, “A priori estimation of the narrow-band UVB phototherapy outcome for moderate-to-severe psoriasis based on the patients’ questionnaire and blood tests using random forest classifier”, (in eng). Clin Cosmet Investig Dermatol. 2021;14:253–9. 10.2147/CCID.S296604. PMC7987278 33776466

[30] Bhutani T, Liao W. “A practical approach to home UVB phototherapy for the treatment of generalized psoriasis”, (in eng). Pract Dermatol. 2010;7(2):31–5. 25191138 PMC4151182

[31] Foulkes AC, “A framework for multi-omic prediction of treatment response to biologic therapy for psoriasis,” (in eng). J Invest Dermatol. 2019;139(1):100–7. 10.1016/j.jid.2018.04.041. 30030151 PMC7239345

[32] Correa da Rosa J, Kim J, Tian S, Tomalin LE, Krueger JG, Suárez-Fariñas M. “Shrinking the psoriasis assessment gap: early gene-expression profiling accurately predicts response to long-term treatment”, (in eng). J Invest Dermatol. 2017;137(2):305–12. 10.1016/j.jid.2016.09.015. 27667537

[33] Tomalin LE, “Early quantification of systemic inflammatory proteins predicts long-term treatment response to tofacitinib and etanercept”, (in eng). J Invest Dermatol. 2020;140(5):1026–34. 10.1016/j.jid.2019.09.023. 31705874

[34] Bagel J, Wang Y, Montgomery P III, Abaya C, Andrade E, Boyce C, Tomich T, Lee B-I, Pariser D, Menter A, Dickerson T. A machine learning-based test for predicting response to psoriasis biologics. Skin J Cutan Med. 2021;5(6):621–38. 10.25251/skin.5.6.5.

[35] Du AX, “Machine learning model for predicting outcomes of biologic therapy in psoriasis”, (in eng). J Am Acad Dermatol. 2023;88(6):1364–7. 10.1016/j.jaad.2022.12.046. 36720368

[36] Orsini C, “Line-field confocal optical coherence tomography: New insights for psoriasis treatment monitoring”, (in eng). J Eur Acad Dermatol Venereol. 2024;38(2):325–31. 10.1111/jdv.19568. 37823360

[37] Reynolds K, Comparing the Quality of ChatGPT- and Physician-Generated Responses to Patients’ Dermatologic Questions in the Electronic Medical Record. Clin Exp Dermatol. 2024;llad456. 10.1093/ced/llad456. Epub ahead of print. 38180108

[38] Mondal H, Mondal S, Podder I. “Using ChatGPT for Writing Articles for Patients’ Education for Dermatological Diseases: A Pilot Study”, (in eng). Indian Dermatol Online J. 2023;14(4):482–6. 10.4103/idoj.idoj_72_23. 37521213 PMC10373821

[39] Zhang Y, “Assessing the ability of an artificial intelligence chatbot to translate dermatopathology reports into patient-friendly language: A cross-sectional study”, (in eng). J Am Acad Dermatol. 2024;90(2):397–9. 10.1016/j.jaad.2023.09.072. 37804932

[40] Chen ML, Rotemberg V, Lester JC, Novoa RA, Chiou AS, Daneshjou R. “Evaluation of diagnosis diversity in artificial intelligence datasets: a scoping review”, (in eng). Br J Dermatol. 2023;188(2):292–4. 10.1093/bjd/ljac047. 36763858

[41] Daneshjou R, Smith MP, Sun MD, Rotemberg V, Zou J. “Lack of Transparency and Potential Bias in Artificial Intelligence Data Sets and Algorithms: A Scoping Review”, (in eng). JAMA Dermatol. 2021;157(11):1362–9. 10.1001/jamadermatol.2021.3129. 34550305 PMC9379852

